# Characterization of antimicrobial and hemolytic properties of short synthetic cationic lipopeptides based on QSAR/QSTR approach

**DOI:** 10.1007/s00726-017-2530-2

**Published:** 2017-12-20

**Authors:** Katarzyna E. Greber, Krzesimir Ciura, Mariusz Belka, Piotr Kawczak, Joanna Nowakowska, Tomasz Bączek, Wiesław Sawicki

**Affiliations:** 1 Department of Physical Chemistry, Faculty of Pharmacy, Medical University of Gdańsk, Al. Gen. J. Hallera 107, 80-416 Gdańsk, Poland; 2 Department of Pharmaceutical Chemistry, Faculty of Pharmacy, Medical University of Gdańsk, Al. Gen. J. Hallera 107, 80-416 Gdańsk, Poland

**Keywords:** QSAR, QSRR, Antimicrobial lipopeptides, MIC, Hemolysis

## Abstract

In this study, we investigated the influence of molecular descriptors of cationic lipopeptides on their antimicrobial activity and hemolytic properties. The quantitative structure–activity relationship and quantitative structure–property relationship models were constructed. The antimicrobial, hemolytic and retention data were used as dependent variable and structural parameters as the independent ones. The obtained results suggest that the chromatographic indexes can be employed for prediction of antibacterial activity and that lipopeptides present nonspecific interaction between erythrocytes and bacterial membranes.

**Electronic supplementary material:**

The online version of this article (10.1007/s00726-017-2530-2) contains supplementary material, which is available to authorized users.

## Introduction

Development of new antimicrobial agents is of the most important challenge these days. Antimicrobial peptides and lipopeptides (AMPs), which reveal serious therapeutic potential due to the broad spectrum of activity, rapid bacterial killing, and synergy with classical antibiotics, are seen to be very promising candidates. The antibacterial mode of action of peptides and lipopeptides is associated mostly with the interactions with bacterial bilayer (Colomb-Cotinat et al. 2016; Greber and Dawgul 2017).

Lipophilicity of compounds is well known as a vital parameter in a quantitative structure–property relationship (QSPR), quantitative structure–activity relationship (QSAR) studies and quantitative structure–toxicity relationship (QSTR). A particular example of QSPR is a quantitative structure–retention relationship (QSRR) where the properties are defined as chromatographic parameters. The QSRR/QSAR approach was successfully applied to predict antimicrobial activities of others class of antibiotics (Ciura et al. 2016).

The main aim of this study was to investigate how molecular descriptors influence the antimicrobial activity and hemolytic properties of short cationic lipopeptides. Additionally, QSRR models were built to evaluate the most important descriptors that influence the chromatographically determined lipophilicity of this class of chemicals.

## Experimental

### Synthesis and purification

Lipopeptides were synthesized, purified and analyzed according to the procedures described in details elsewhere (Greber et al. 2017).

### MIC and MHC

The minimum inhibitory concentration (MIC) was determined according to the procedure recommended by the [Clinical Laboratory Standards Institute (CLSI) (2012, 2017)]. The following Gram-positive strains were used: *Staphylococcus aureus* (ATCC 25923), *S. epidermidis* (PCM 2118), *Bacillus subtilis* (ATCC 6633), and *Enterococcus faecalis* (ATCC 29212). Minimum hemolytic concentration (MHC) was taken as the lowest concentration of lipopeptides which induced 10% of hemolysis of human red blood cells. Antimicrobial activity (MIC) toward Gram-positive strains and toxicity toward human red blood cells (MHC) are presented in supplementary materials in Table 1S.

### Chromatographic analysis

The RP-HPLC experiments were performed on Shimadzu Prominence apparatus on a Chromolith^®^ Performance RP-18 endcapped 100–4.6 mm monolithic column with a linear gradient 2–98% phase B (where phase A was 0.1% TFA in water and phase B was 0.1% TFA in ACN), at a flow rate of 2 mL/min, and UV detection at 214 nm. The concentrations of lipopeptide samples were 100 µg/mL and the injected volume was 10 µL (Greber et al. 2017).

### Molecular modeling

HyperChem 8.08 (Hypercube, Waterloo, Canada) software was used for the calculation of molecular descriptors. The preliminary optimization of investigated compounds was carried out using the molecular mechanic calculations (MM+). In the next step, semi-empirical calculation method Austin Model 1 (AM1) was applied (HyperChem Computational Chemistry 1996). After calculation of molecular structures, Dragon 7.0 (Talete, Milan, Italy) software was used to calculate further set of constitutional indices, ring descriptors, the functional group counts, atom-centered fragments, atom-type E-state indices, CATS 2D, 2D Atom Pairs, molecular properties and charge descriptors (Dragon 7 molecular descriptors 2017; odeschini and onsonni 2009). Finally, 162 descriptors were used for analysis.

### QSAR/QSTR/QSRR analysis

For the construction of QSAR, QSRR and QSTR models, multiple linear regression (MLR), partial least squares (PLS) and orthogonal partial least squares (OPLS) were applied (Roy et al. 2015; Worley and Powers 2013; Saxena and Prathipati 2003). During calculation, the log MIC value, log MHC and retention data (log_*k*_) were used as dependent variable and structural parameters as the independent ones. In case of MLR calculation stepwise regression mode was chosen. This calculation was performed on Statistica software (Statistica 12, Statsoft, USA). The coefficient of correlation (*r*) and determination (*R* ^2^), *F* test value, standard deviation and the standard estimation error were used as the bases for testing the established MLR model. Next, using Simca software (Simca 13, Umetrix, Umea, Sweden) PLS and OLPS models were constructed (User Guide to SIMCA 2017). The validation of the established models was performed with leave-one out procedure based on *Q* ^2^ value (Alexander et al. 2015).

## Results and discussion

Although the antimicrobial peptides are concerned as potential drugs, their mechanism of action is still not fully known. Several models have been proposed for last decades, including pore formation (Brogden 2005), detergent-like permeabilization of the bilayer (Bechinger and Lohner 2006), and membrane destabilization after AMPs coat the bilayer surface (Shai and Oren 2001). Judging the proposed models, it seems likely that there is no single mechanism which can explain AMP mechanism of action. Probably, AMPs of different chemical origins may be described by one or more of the above models (Horn et al. 2012). For this reason, the identification of the most important physicochemical descriptors, which affect the antimicrobial activities, is useful to gather the knowledge how the investigated class of AMPs works.

**Fig. 1:**
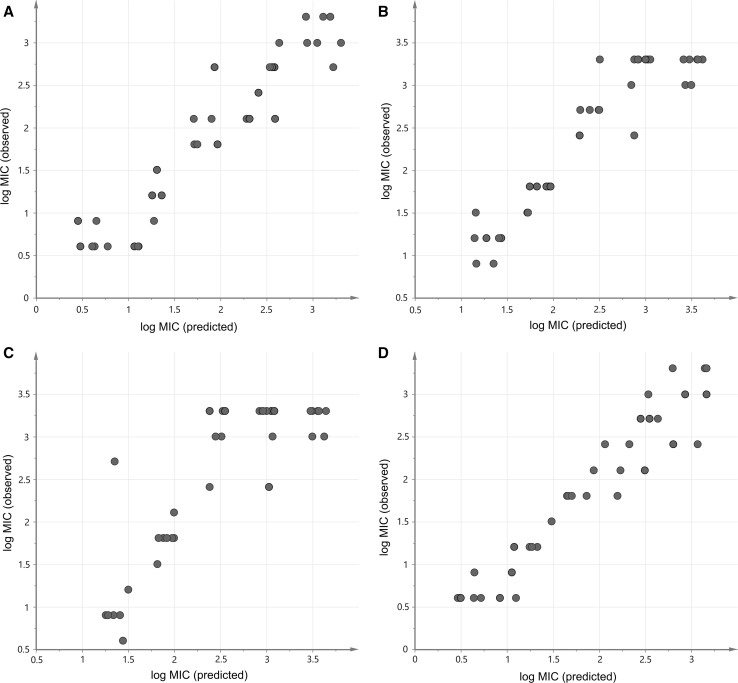
The comparison of performance for the obtained QSAR models for each strain of bacteria: **a** *Bacillus subtilis*, **b** *Staphylococcus aureus*, **c** *Staphylococcus epidermidis* and **d** *Enterococcus faecalis*

The dataset that includes calculated descriptors and chromatographic parameter log_*k*_ was used for QSAR analysis. Three regression methods MLR, PLS and OPLS were tested. Although both PLS and OPLS can be used for analysis of highly collinear data, the advantage of OPLS method in compression of PLS is an integrated orthogonal signal correction filter. The best QSAR models are obtained after OPLS calculation. The fifteen most important descriptors are listed in Table 1. All obtained models meet the Tropsha et al. (2003) criteria (*R* ^2^ > 0.6 and *Q* ^2^ > 0.5). It is worth to notice that all obtained models are based practically on the same descriptors. This finding suggested that the mechanism of action against Gram-positive bacteria is nonspecific. The differences of MIC values obtained for each type of bacteria can be explained by the different affinity of bacterial membranes. The composition of lipid bilayer could be the main factor, which determines the higher activity of lipopeptides toward *Staphylococcus epidermidis*, and *Bacillus subtilis* than toward *Staphylococcus aureus*. The investigated lipopeptides showed the lowest activity against *Enterococcus faecalis*. In Table 2S, the lipidome map of tested strains is presented. The concentration of phosphatidylglycerols (PG), the negatively charged phospholipids, seems to be the major factor of interaction with lipopeptides. In case of *S. epidermidis*, *B. subtilis* concentration of PG is similar (67 vs 70%), and the observed MIC values are the lowest. On the other hand, the *E. faecalis* membrane contains only 20% PG and the MIC values are the highest. Whereas in case of *S. aureus* the percent of PG in the membrane is 40%, so it is moderate among tested microbes, and also the moderate activity of lipopeptides were noticed (Fig. 1).

**Table 1:** List of molecular descriptors characterized by the highest VIP values in OPLS models built for QSAR models and QSTR model

Descriptor	*R* ^2^ = 0.949	*Q* ^2^ = 0.890	
	VIP	Full name	Block
*Bacillus subtilis*			
CATS2D_03_LL	2.91	CATS (chemically advanced template search) 2D Lipophilic–Lipophilic at lag 03	CATS 2D
CATS2D_04_LL	2.91	CATS2D Lipophilic–Lipophilic at lag 04	CATS 2D
H-046	2.83	H attached to C0(sp3) no × attached to next C	Atom-centered fragments
CATS2D_02_LL	2.83	CATS2D Lipophilic–Lipophilic at lag 02	CATS 2D
CATS2D_05_LL	2.81	CATS2D Lipophilic–Lipophilic at lag 05	CATS 2D
SssCH2	2.79	Sum of ssCH2 E-states	Atom-type E-state indices
SsCH3	2.75	Sum of ssCH3 E-states	Atom-type E-state indices
ALOGP	2.66	Ghose–Crippen octanol–water partition coeff. (log *P*)	Molecular properties
ALOGP2	2.53	Squared Ghose–Crippen octanol–water partition coeff. (log *P*^2)	Molecular properties
CATS2D_01_LL	2.52	CATS2D Lipophilic–Lipophilic at lag 01	CATS 2D
CATS2D_06_LL	2.44	CATS2D Lipophilic–Lipophilic at lag 06	CATS 2D
Log_*k*_	2.38	HPLC retention factor	Experimental
C-002	2.17	CH2R2	Atom-centred fragments
CATS2D_00_LL	2.17	CATS2D Lipophilic–Lipophilic at lag 00	CATS 2D
CATS2D_07_LL	1.95	CATS2D Lipophilic–Lipophilic at lag 07	CATS 2D

Descriptor	*R* ^2^ = 0.949	*Q* ^2^ = 0.860	
	VIP	Full name	Block
*Enterococcus faecalis*			
CATS2D_03_LL	3.11	CATS2D Lipophilic–Lipophilic at lag 03	CATS 2D
CATS2D_04_LL	3.11	CATS2D Lipophilic–Lipophilic at lag 04	CATS 2D
ALOGP2	2.95	Squared Ghose–Crippen octanol–water partition coeff. (log *P*^2)	Molecular properties
ALOGP	2.95	Ghose–Crippen octanol–water partition coeff. (log *P*)	Molecular properties
H-046	2.85	H attached to C0(sp3) no × attached to next	Atom-centred fragments
CATS2D_02_LL	2.85	CATS2D Lipophilic–Lipophilic at lag 02	CATS 2D
SsCH3	2.84	Sum of ssCH3 E-states	Atom-type E-state indices
CATS2D_05_LL	2.84	CATS2D Lipophilic–Lipophilic at lag 05	CATS 2D
SssCH2	2.78	Sum of ssCH2 E-states	Atom-type E-state indices
Log_*k*_	2.76	HPLC retention factor	
CATS2D_01_LL	2.39	CATS2D Lipophilic–Lipophilic at lag 01	CATS 2D
CATS2D_06_LL	2.33	CATS2D Lipophilic–Lipophilic at lag 06	CATS 2D
BLTD48	2.18	Verhaar Daphnia base-line toxicity from MLOGP (mmol/L)	Molecular properties
BLTF96	2.18	Verhaar Fish base-line toxicity from MLOGP (mmol/L)	Molecular properties
MLOGP	2.18	Moriguchi octanol–water partition coeff. (log *P*)	Molecular properties

Descriptor	*R* ^2^ = 0.949	*Q* ^2^ = 0.681	
	VIP	Full name	Block
*Staphylococcus aureus*			
CATS2D_03_LL	3.13	CATS2D Lipophilic–Lipophilic at lag 03	CATS 2D
CATS2D_04_LL	3.13	CATS2D Lipophilic–Lipophilic at lag 04	CATS 2D
H-046	2.92	H attached to C0(sp3) no × attached to next	Atom-centred fragments
CATS2D_02_LL	2.92	CATS2D Lipophilic–Lipophilic at lag 02	CATS 2D
ALOGP	2.92	Ghose–Crippen octanol–water partition coeff. (log *P*)	Molecular properties
CATS2D_05_LL	2.91	CATS2D Lipophilic–Lipophilic at lag 05	CATS 2D
ALOGP2	2.89	squared Ghose–Crippen octanol–water partition coeff. (log *P*^2)	Molecular properties
SssCH2	2.85	Sum of ssCH2 E-states	Atom-type E-state indices
SsCH3	2.79	Sum of ssCH3 E-states	Atom-type E-state indices
Log_*k*_	2.72	HPLC retention factor	Experimental
CATS2D_01_LL	2.49	CATS2D Lipophilic–Lipophilic at lag 01	CATS 2D
CATS2D_06_LL	2.43	CATS2D Lipophilic–Lipophilic at lag 06	CATS 2D
C-002	2.06	CH2R2	Atom-centred fragments
CATS2D_00_LL	2.06	CATS2D Lipophilic–Lipophilic at lag 00	CATS 2D
BLTF96	2.04	Verhaar Fish base-line toxicity from MLOGP (mmol/L)	Molecular properties

Descriptor	*R* ^2^ = 0.948	*Q* ^2^ = 0.898	
	VIP	Full name	Block
*Staphylococcus epidermidis*			
CATS2D_03_LL	2.83	CATS2D Lipophilic–Lipophilic at lag 03	CATS 2D
CATS2D_04_LL	2.83	CATS2D Lipophilic–Lipophilic at lag 04	CATS 2D
H-046	2.79	H attached to C0(sp3) no × attached to next	Atom-centred fragments
CATS2D_02_LL	2.79	CATS2D Lipophilic–Lipophilic at lag 02	CATS 2D
SssCH2	2.78	Sum of ssCH2 E-states	Atom-type E-state indices
CATS2D_05_LL	2.77	CATS2D Lipophilic–Lipophilic at lag 05	CATS 2D
SsCH3	2.66	Sum of ssCH3 E-states	Atom-type E-state indices
ALOGP	2.64	Ghose–Crippen octanol–water partition coeff. (log *P*)	Molecular properties
ALOGP2	2.56	squared Ghose–Crippen octanol–water partition coeff. (log *P*^2)	Molecular properties
CATS2D_01_LL	2.51	CATS2D Lipophilic–Lipophilic at lag 01	CATS 2D
CATS2D_06_LL	2.44	CATS2D Lipophilic–Lipophilic at lag 06	CATS 2D
Log_*k*_	2.24	HPLC retention factor	Experimental
C-002	2.18	CH2R2	Atom-centred fragments
CATS2D_00_LL	2.18	CATS2D Lipophilic–Lipophilic at lag 00	CATS 2D
CATS2D_07_LL	1.98	CATS2D Lipophilic–Lipophilic at lag 07	CATS 2D

1 + 2+0	*R* ^2^ = 0.949	*Q* ^2^ = 0.841	
Descriptor	VIP	Full name	Block
*QSTR*			
CATS2D_03_LL	3.12	CATS2D Lipophilic–Lipophilic at lag 00	CATS 2D
CATS2D_04_LL	3.12	CATS2D Lipophilic–Lipophilic at lag 04	CATS 2D
ALOGP2	3.00	Squared Ghose–Crippen octanol–water partition coeff. (log *P*^2)	Molecular properties
ALOGP	2.94	Ghose–Crippen octanol–water partition coeff. (log *P*)	Molecular properties
H-046	2.88	H attached to C0(sp3) no × attached to next	Atom-centred fragments
CATS2D_02_LL	2.88	CATS2D Lipophilic–Lipophilic at lag 02	CATS 2D
CATS2D_05_LL	2.86	CATS2D Lipophilic–Lipophilic at lag 05	CATS 2D
SssCH2	2.82	Sum of ssCH2 E-states	Atom-type E-state indices
SsCH3	2.79	Sum of ssCH3 E-states	Atom-type E-state indices
Log_*k*_	2.71	HPLC retention factor	
CATS2D_01_LL	2.43	CATS2D Lipophilic–Lipophilic at lag 01	CATS 2D
CATS2D_06_LL	2.35	CATS2D Lipophilic–Lipophilic at lag 06	CATS 2D
BLTF96	2.14	Verhaar Fish base-line toxicity from MLOGP (mmol/L)	Molecular properties
BLTD48	2.14	Verhaar Daphnia base-line toxicity from MLOGP (mmol/L)	Molecular properties
MLOGP	2.14	Moriguchi octanol–water partition coeff. (log *P*)	Molecular properties

*R* ^2^ denotes coefficient of determination for the model, *Q* ^2^ denotes cross-validated coefficient of determination for the model

When we look inside of the obtained QSAR models, additional conclusions can be drawn. The most important descriptors used for building of OPLS models are the same in their nature. They are related to lipophilicity properties, such as CATS descriptors, Ghose–Crippen octanol–water partition coefficient (ALOGP) as well as the number of C atoms. The special attraction of CATS descriptors is its exhaustive 2D pharmacophore/biophore model based on the cross-correlation of generalized atom types (Schneider et al. 1999). Its usefulness for QSAR studies indicated several reports (Ahmed et al. 2013; Reutlinger et al. 2013). Furthermore, the chromatographically obtained parameters (log_*k*_), which can be interpreted as chromatographic lipophilicity index, have a similar impact like calculated lipophilicity.

This finding highly indicated that log_*k*_ reflects lipophilic properties of lipopeptides and can be concerned as log *P* surrogate. The traditional scales of lipophilicity is based on partition coefficient between two phases, *n*-octanol and water, a system that is conventionally used due to its partitioning analogy with the biological environment. However, the traditional approach (so-called shake flask method) has significant limitations. It is laborious, time-consuming, requires pure substances in large quantities. Moreover, the compounds which exhibit surface-active properties, as investigated lipopeptides, cannot be analyzed in this way. Therefore, the chromatographic approach was used to assess lipophilicity of this class of chemical derivatives. To gain more insight into molecular mechanism of retention, the QSRR approach was used. The lipophilicity index measured by HPLC is derived by the retention time that is converted to the logarithm of the retention factor log_*k*_ (Dreher et al. 2017). The “one-run gradient method” was describes in the literature as an attractive alternative to performing several isocratic runs followed by extrapolation (Giaginis and Tsantili-Kakoulidou 2008; Liang et al. 2017).

As a means to investigate the relationship between molecular properties and retention, firstly the MLR regression was applied. The best MLR model that includes three descriptors (sum of sCH3 E-states [SsCH3], sum of sNH2 E-states [SsNH2] and frequency of C–C at topological distance 9 [F09[C–C]]), is presented below:

$$ \log_{k} = - \;2.544\;\left( { \pm \;0.492} \right) + 1.589\;\left( { \pm \; 0.212} \right){\text{SsCH}}_{ 3} - 0.029\;\left( { \pm \;0.005} \right){\text{SsNH}}_{ 2} + 0.012\;\left( { \pm \; 0.002} \right){\text{F}}09\left[ {{\text{C}} - {\text{C}}} \right] $$

**Table:** 

1.32 × 10^−5^	1.97 × 10^−8^	1.36 × 10^−5^	2.10 × 10^−4^

*R* = 0.955	*R* ^2^ = 0.913	*F* = 107.656	*s* = 0.034	*p* = 1.96 × 10^−14^

As might be expected the increased number of C atoms in carbon chain leads to increased retention. Oppositely, sum of sNH2 E-states, a group that influences the polarity of the molecule, reduces retention of investigated lipopeptides. The NH_2_ group can be responsible for interaction with polar mobile phase. The result of PLS and OPLS regression analysis are presented in Table 3S. The statistical parameters of all obtained QSRR models are similar. The most important factors, according to VIP value are listed in Table 3S. The descriptors that highly influence the value of chromatographic parameter log_*k*_ are connected with calculated lipophilicity (ALOGP and MLOGP descriptors but also CATS descriptors) and the number of C atoms in a molecule. Oppositely to MLR model, the influence of NH_2_ group was not underlined in the obtained PLS and OPLS models. However, the calculated lipophilicity indexes can include this information, since the MLOG and ALOGP calculation algorithms use the whole structure and all functional groups of a molecule. Summarizing the QSRR analysis, the log_*k*_ parameter reflects very well with lipophilic properties of investigated lipopeptides.

The last step of our study concerned QSTR. One of the factors limiting the clinical use of lipopeptides is their hemolytic characters. Although, the coarse-grained molecular dynamics simulations revealed no association between the lipopeptides and model mammalian bilayers, the hemolytic properties of lipopeptides were previously reported (Greber et al. 2017). It should be noticed that the hemolytic concentration of lipopeptides is significantly higher as antimicrobial, but it still limits clinical use of AMPs. The obtained QSTR models (Table 1) suggested that lipopeptides degrade cell membranes of erythrocytes in the same way as bacterial membranes. Descriptors obtained in the QSTR–OPLS model are very similar to those previously described in QSAR models, belong to the same class and they are connected with lipophilic properties of target compounds.

## Conclusion

The obtained results suggested that the simple HPLC method could be used for lipophilicity assessment of short cationic lipopeptides. Furthermore, the chromatographic indexes can be useful for prediction of antibacterial activity. Summarizing, the QSAR and QSTR analysis, all obtained models indicate that lipophilicity play vital role. This result is not surprising since lipophilicity is well known as the physicochemical parameter that determines biological properties of xenobiotics. The most important conclusion is the fact that lipopeptides show a nonspecific interaction between erythrocytes and bacterial membranes. Different affinities between mammalian and bacterial bilayers seem to be the vital point to design more active and less toxic antimicrobial lipopeptides.

## Electronic supplementary material

Below is the link to the electronic supplementary material.

Supplementary material 1 (DOCX 31 kb)
