# Supplementary material for: Characterization of antimicrobial and hemolytic properties of short synthetic cationic lipopeptides based on QSAR/QSTR approach
Source: Amino Acids. 2017 Dec 20;50(3):479–85. doi: 10.1007/s00726-017-2530-2 (PMC5852172; doi:10.1007/s00726-017-2530-2)
Supplement: Supplementary file 1 — Supplementary material 1 (DOCX 31 kb) [file 726_2017_2530_MOESM1_ESM.docx]

**Supplementary data**

**Characterization of antimicrobial and hemolytic properties of short synthetic cationic lipopeptides based on QSAR/QSTR approach**

Katarzyna E. Greber^1^*^&^, Krzesimir Ciura^1&^, Mariusz Belka^2^ , Piotr Kawczak^2^, Joanna Nowakowska^1^,Tomasz Bączek^2^, Wiesław Sawicki^1^

^1^ Medical University of Gdansk, Faculty of Pharmacy, Department of Physical Chemistry, Al. Gen. J. Hallera 107, 80-416, Gdansk, Poland

^2^ Medical University of Gdansk, Faculty of Pharmacy, Department of Pharmaceutical Chemistry, Al. Gen. J. Hallera 107, 80-416 Gdansk, Poland

- Corresponding author

Katarzyna E. Greber

E-mail: [greber@gumed.edu.pl](mailto:greber@gumed.edu.pl)
Medical University of Gdansk
Faculty of Pharmacy, Department of Physical Chemistry
Al. Gen. J. Hallera 107
80-416, Gdansk, Poland

^&^ These authors contributed equally to this work

**Table 1S.** Antimicrobial activity toward Gram-positive strains and toxicity toward human red blood cells [1].

| **Lipopeptides** | ***Staphylococcus aureus*** | ***Staphylococcus epidermidis*** | ***Bacillus subtillis*** | ***Enterococcus fecalis*** | **Haemolysis** |
| --- | --- | --- | --- | --- | --- |
|  | MIC [µg/mL] | | | | MHC [µg/mL] |
| C_16_-K-NH_2_ | >512 | 8 | 8 | 32 | 512 |
| C_16_-KK-NH_2_ | 8 | 4 | 4 | 8 | 256 |
| C_16_-KKK-NH_2_ | 8 | 4 | 8 | 16 | 32 |
| C_16_-KKKK-NH_2_ | 4 | 4 | 4 | 16 | 16 |
| C_16_-KG-NH_2_ | 8 | 4 | 4 | 16 | 256 |
| C_16_-KGK-NH_2_ | 8 | 4 | 4 | 8 | 64 |
| C_16_-KGKG-NH_2_ | 16 | 4 | 4 | 16 | 64 |
| C_14_-K-NH_2_ | 64 | 16 | 32 | 32 | 512 |
| C_14_-KK-NH_2_ | 64 | 4 | 4 | 32 | 128 |
| C_14_-KKK-NH_2_ | 64 | 8 | 4 | 64 | 256 |
| C_14_-KKKK-NH_2_ | 64/128 | 16 | 4 | 64 | 256 |
| C_14_-KG-NH_2_ | 32 | 16 | 16 | 32 | >1·10^3^ |
| C_14_-KGK-NH_2_ | 64 | 16 | 8 | 64 | 128 |
| C_14_-KGKG-NH_2_ | 64 | 32 | 16 | 64 | 512 |
| C_12_-K-NH_2_ | 256 | 128 | 128 | 256 | 1·10^3^ |
| C_12_-KK-NH_2_ | 2·10^3^ | 64 | 64 | 512 | >4·10^3^ |
| C_12_-KKK-NH_2_ | 1·10^3^ | 64 | 64 | 512 | 512 |
| C_12_-KKKK-NH_2_ | 1·10^3^ | 64 | 128 | 512 | 1·10^3^ |
| C_12_-KG-NH_2_ | 256 | 128 | 128 | 256 | 1·10^3^ |
| C_12_-KGK-NH_2_ | >2·10^3^ | 64 | 512 | 2·10^3^ | 1·10^3^ |
| C_12_-KGKG-NH_2_ | 2·10^3^ | 256 | 64 | 512 | 256 |
| C_10_-K-NH_2_ | 1·10^3^ | 1·10^3^ | 1·10^3^ | >2·10^3^ | >4·10^3^ |
| C_10_-KK-NH_2_ | >2·10^3^ | 256 | 256 | >2·10^3^ | 4·10^3^ |
| C_10_-KKK-NH_2_ | >2·10^3^ | 128 | 128 | >2·10^3^ | >4·10^3^ |
| C_10_-KKKK-NH_2_ | >2·10^3^ | 64 | 128 | >2·10^3^ | >4·10^3^ |
| C_10_-KG-NH_2_ | >2·10^3^ | 512 | 512 | 1·10^3^ | >4·10^3^ |
| C_10_-KGK-NH_2_ | >2·10^3^ | 512 | 512 | >2·10^3^ | >4·10^3^ |
| C_10_-KGKG-NH_2_ | >2·10^3^ | 512 | 512 | 2·10^3^ | >4·10^3^ |
| C_8_-K-NH_2_ | >1·10^3^ | >1·10^3^ | >1·10^3^ | >1·10^3^ | >4·10^3^ |
| C_8_-KK-NH_2_ | >1·10^3^ | >1·10^3^ | >1·10^3^ | >1·10^3^ | >4·10^3^ |
| C_8_-KKK-NH_2_ | >2·10^3^ | >256 | 1·10^3^ | >2·10^3^ | >4·10^3^ |
| C_8_-KKKK-NH_2_ | >2·10^3^ | >2·10^3^ | >2·10^3^ | 2·10^3^ | >4·10^3^ |
| C_8_-KG-NH_2_ | >2·10^3^ | >2·10^3^ | >2·10^3^ | >2·10^3^ | >4·10^3^ |
| C_8_-KGK-NH_2_ | >2·10^3^ | 256 | 512 | >2·10^3^ | >4·10^3^ |
| C_8_-KGKG-NH_2_ | >2·10^3^ | >2·10^3^ | >2·10^3^ | >2·10^3^ | >4·10^3^ |

**Table 2S.** The main lipid classes of bacterial strains used in the study.

| Strain | Weight Percentage of Total Lipid of Cell Membrane | | | | | | | References |
| --- | --- | --- | --- | --- | --- | --- | --- | --- |
|  | Negatively charged | | Uncharged | | | Positively charged | **Other lipids** |  |
|  | **PG** | **GPDGDG** | **DGDG** | **PE** | **CL** | **LPG** |  |  |
| *Staphylococcus aureus* | 43 | - | - | - | 22 | 30 | 5 | [2] |
| *Staphylococcus epidermidis* | 67 | 5 | 20 | - | - | - | 8 | [3] |
| *Bacillus subtilis* | 70 | - | - | 12 | 4 | - | 14 | [4] |
| *Enterococcus faecalis* | 20 | 5 | - | - | 44 | 6 | 25 | [5] |

PG – glycerophosphoglycerols

GPDGDG – glycerophosphoglycosyldiacylglycerols

DGDG – glycosyldiacylglycerols

PE – glycerophosphoethanolamines

CL – diacylglycerophosphoglycerophosphodiradylglycerols

LPG – lysyl-glycerophosphoglycerols

**Table 3S.** Results of PLS and OPLS analysis with the list of fifteen molecular descriptors characterized by the highest VIP values in obtained QSRR models built for investigated chromatographic systems.

| PLS | | | | | | |
| --- | --- | --- | --- | --- | --- | --- |
| R^2^=0.967 | | | Q^2^=0.893 | A=4 | |  |
| Descriptor | VIP | Full name | | | Block | |
| CATS2D_03_LL | 2.32 | CATS2D Lipophilic-Lipophilic at lag 03 | | | CATS 2D | |
| CATS2D_04_LL | 2.32 | CATS2D Lipophilic-Lipophilic at lag 04 | | | CATS 2D | |
| SsCH3 | 2.18 | Sum of ssCH3 E-states | | | Atom-type E-state indices | |
| H-046 | 2.14 | H attached to C0(sp3) no X attached to next | | | Atom-centred fragments | |
| CATS2D_02_LL | 2.14 | CATS2D Lipophilic-Lipophilic at lag 02 | | | CATS 2D | |
| CATS2D_05_LL | 2.06 | CATS2D Lipophilic-Lipophilic at lag 05 | | | CATS 2D | |
| SssCH2 | 2.00 | Sum of ssCH2 E-states | | | Atom-type E-state indices | |
| ALOGP | 1.98 | Ghose-Crippen octanol-water partition coeff. (logP) | | | Molecular properties | |
| ALOGP2 | 1.95 | squared Ghose-Crippen octanol-water partition coeff. (logP^2) | | | Molecular properties | |
| CATS2D_01_LL | 1.84 | CATS2D Lipophilic-Lipophilic at lag 01 | | | CATS 2D | |
| CATS2D_06_LL | 1.69 | CATS2D Lipophilic-Lipophilic at lag 06 | | | CATS 2D | |
| C-002 | 1.55 | CH2R2 | | | Atom-centred fragments | |
| CATS2D_00_LL | 1.55 | CATS2D Lipophilic-Lipophilic at lag 00 | | | CATS 2D | |
| NssCH2 | 1.44 | Number of atoms of type ssCH2 | | | Atom-type E-state indices | |
| BLTF96 | 1.40 | Verhaar Fish base-line toxicity from MLOGP (mmol/l) | | | Molecular properties | |
| OPLS | | | | | | |
| R^2^=0.967 | Q^2^=0.897 | 1 + 3 + 0 | | |  | |
| Descriptor | VIP | Full name | | | Block | |
| SsCH3 | 2.19 | Sum of ssCH3 E-states | | | Atom-type E-state indices | |
| CATS2D_03_LL | 2.12 | CATS2D Lipophilic-Lipophilic at lag 03 | | | CATS 2D | |
| CATS2D_04_LL | 2.12 | CATS2D Lipophilic-Lipophilic at lag 04 | | | CATS 2D | |
| ALOGP | 2.12 | Ghose-Crippen octanol-water partition coeff. (logP) | | | Molecular properties | |
| ALOGP2 | 2.08 | squared Ghose-Crippen octanol-water partition coeff. (logP^2) | | | Molecular properties | |
| BLTF96 | 1.90 | Verhaar Fish base-line toxicity from MLOGP (mmol/l) | | | Molecular properties | |
| BLTD48 | 1.90 | Verhaar Daphnia base-line toxicity from MLOGP (mmol/l) | | | Molecular properties | |
| MLOGP | 1.90 | Moriguchi octanol-water partition coeff. (logP) | | | Molecular properties | |
| BLTA96 | 1.90 | Verhaar Algae base-line toxicity from MLOGP (mmol/l) | | | Molecular properties | |
| MLOGP2 | 1.85 | squared Moriguchi octanol-water partition coeff. (logP^2) | | | Molecular properties | |
| H-046 | 1.63 | H attached to C0(sp3) no X attached to next | | | Atom-centred fragments | |
| CATS2D_02_LL | 1.63 | CATS2D Lipophilic-Lipophilic at lag 02 | | | CATS 2D | |
| CATS2D_05_LL | 1.59 | CATS2D Lipophilic-Lipophilic at lag 05 | | | CATS 2D | |
| SssCH2 | 1.50 | Sum of ssCH2 E-states | | | Atom-type E-state indices | |
| Hy | 1.20 | hydrophilic factor | | | Molecular properties | |

**References**

1. Greber KE, Dawgul M, Kamysz W, Sawicki W (2017) Cationic Net Charge and Counter Ion Type as Antimicrobial Activity Determinant Factors of Short Lipopeptides. Front. Microbiol. 8:123. doi: 10.3389/fmicb.2017.00123.
2. [Hayami M](https://www.ncbi.nlm.nih.gov/pubmed/?term=Hayami%2520M%255BAuthor%255D&cauthor=true&cauthor_uid=491988), [Okabe A](https://www.ncbi.nlm.nih.gov/pubmed/?term=Okabe%2520A%255BAuthor%255D&cauthor=true&cauthor_uid=491988), [Kariyama R](https://www.ncbi.nlm.nih.gov/pubmed/?term=Kariyama%2520R%255BAuthor%255D&cauthor=true&cauthor_uid=491988), [Abe M](https://www.ncbi.nlm.nih.gov/pubmed/?term=Abe%2520M%255BAuthor%255D&cauthor=true&cauthor_uid=491988), [Kanemasa Y](https://www.ncbi.nlm.nih.gov/pubmed/?term=Kanemasa%2520Y%255BAuthor%255D&cauthor=true&cauthor_uid=491988) (1979) Lipid composition of Staphylococcus aureus and its derived L-forms. Microbiol Immunol. 23:435-442.
3. [Komaratat P](https://www.ncbi.nlm.nih.gov/pubmed/?term=Komaratat%2520P%255BAuthor%255D&cauthor=true&cauthor_uid=1174526), [Kates M](https://www.ncbi.nlm.nih.gov/pubmed/?term=Kates%2520M%255BAuthor%255D&cauthor=true&cauthor_uid=1174526) (1975) The lipid composition of a halotolerant species of Staphylococcus epidermidis. Biochim Biophys Acta. 398:464-484.
4. Clejan S, Krulwich TA, Mondrus KR, Seto-Young D (1986) Membrane Lipid Composition of Obligately and Facultatively Alkalophilic Strains of Bacillus spp. J Bacteriol. 168:334–340.
5. Rashid R, Cazenave-Gassiot A, Gao IH, Nair ZJ, Kumar JK, Gao L, Kline KA, Wenk MR (2017) Comprehensive analysis of phospholipids and glycolipids in the opportunistic pathogen Enterococcus faecalis. PLoS One. 12(4): e0175886. doi: 10.1371/journal.pone.0175886.
